# Nicorandil Inhibits Osteoclast Formation Base on NF-κB and p-38 MAPK Signaling Pathways and Relieves Ovariectomy-Induced Bone Loss

**DOI:** 10.3389/fphar.2021.726361

**Published:** 2021-09-08

**Authors:** Shenggui Xu, Xiankun Cao, Zhenxing Yu, Wenxin He, Yichuan Pang, Wang Lin, Zhiqian Chen, Weizhong Guo, Xiongwei Lu, Chengshou Lin

**Affiliations:** ^1^ Department of Orthopaedics, Mindong Hospital Affiliated to Fujian Medical University, Fuan, China; ^2^ Shanghai Key Laboratory of Orthopedic Implants, Department of Orthopaedics Surgery, Shanghai Ninth People’s Hospital, Shanghai Jiaotong University School of Medicine, Shanghai, China; ^3^ Shanghai Key Laboratory of Stomatology, Department of Oral Surgery, National Clinical Research Center of Stomatology, Shanghai Ninth People’s Hospital, Shanghai Jiaotong University School of Medicine, Shanghai, China

**Keywords:** nicorandil, osteoclast, nuclear factor kappa-B, phospho-p38 (p-p38), ovariectomy, osteoporosis

## Abstract

Osteolytic bone disorders are characterized by an overall reduction in bone mineral density which enhances bone ductility and vulnerability to fractures. This disorder is primarily associated with superabundant osteoclast formation and bone resorption activity. Nicorandil (NIC) is a vasodilatory anti-anginal drug with ATP-dependent potassium (K_ATP_) channel openings. However, NIC is adopted to manage adverse cardiovascular and coronary events. Recent research has demonstrated that NIC also possesses anti-inflammatory peculiarity through the regulation of p38 MAPK and NF-κB signaling pathways. Both MAPK and NF-κB signaling pathways play pivotal roles in RANKL-induced osteoclast formation and bone resorption function. Herein, we hypothesized that NIC may exert potential biological effects against osteoclasts, and revealed that NIC dose-dependently suppressed bone marrow macrophage (BMM) precursors to differentiate into TRAP + multinucleated osteoclasts *in vitro*. Furthermore, osteoclast resorption assays demonstrated anti-resorptive effects exhibited by NIC. NIC had no impact on osteoblast differentiation or mineralization function. Based on Biochemical analyses, NIC relieved RANKL-induced ERK, NF-κB and p38 MAPK signaling without noticeable effects on JNK MAPK activation. However, the attenuation of NF-κB and p38 MAPK activation was sufficient to hamper the downstream induction of c-Fos and NFATc1 expression. Meanwhile, NIC administration markedly protected mice from ovariectomy (OVX)-induced bone loss through *in vivo* inhibition of osteoclast formation and bone resorption activity. Collectively, this work demonstrated the potential of NIC in the management of osteolytic bone disorders mediated by osteoclasts.

## Introduction

With the ageing population, numerous bone-related diseases and morbidity including osteoporosis are increasingly becoming a worldwide public health concern. This is attributed to the substantial bone-associated morbidities and mortality, associated socio-economic and healthcare costs (Harvey et al., 2010; Rachner et al., 2011; Yedavally-Yellayi et al., 2019a). Excessive osteoclast-mediated bone resorption underlies the pathological manifestations of bone loss characterized by thinning of cortical bones, reduction in trabecular bone mass and an overall reduction in bone mineral density. These events contribute to an overall deterioration in bone microarchitecture and high risks of bone fractures (Del Puente et al., 2015).

The formation of multinucleated osteoclast transpires from the fusion between mononuclear precursor cells of the macrophage/monocyte lineage in response to two key cytokines, macrophage colony-stimulating factor (M-CSF) and nuclear factor-κB (NF-κB) ligand (RANKL) (Yasuda et al., 1998; Qi et al., 2017). M-CSF drives the initial step to promote precursor cell survival and proliferation, subsequently enhancing the expression of the RANK receptor on the surface of cells (Dougall et al., 1999). Besides, RANKL is the foremost cytokine that drives committed precursors towards the osteoclast lineage (Kong et al., 1999). The binding of RANKL to its sole receptor RANK recruit tumor necrosis factor receptor-associated factor 6 (TRAF6) and activate an array of downstream signaling cascades that induce cellular differentiation and fusion (Sitara and Aliprantis, 2011). Signaling cascades activated by RANKL include NF-κB, extracellular signal-regulated kinase (ERK), and mitogen-activated protein kinases (MAPKs) comprising p38, Akt, and c-jun N-terminal kinase (JNK), leading to the activation of nuclear factor of activated T-cells cytoplasmic 1 (NFATc1) and the transcription factors c-Fos (Wong et al., 1994; Matsumoto et al., 2000; David et al., 2002; Li et al., 2002; Takayanagi et al., 2002; Huang et al., 2006; He et al., 2011; Moon et al., 2012). Therefore, pharmacological chemicals capable of inhibiting RANKL-RANK signaling transmission have great potential in the therapeutical management of osteolytic diseases.

Nicorandil (NIC), a nicotinamide nitrate compound, has agonistic effects against ATP-dependent potassium (K_ATP_) channels. It is clinically used as a vasodilatory drug to treat angina and other cardiovascular and coronary events (Sahara et al., 2012; He et al., 2018). In recent years, accumulating studies have demonstrated the benefits associated with the use of NIC in the management of bronchial asthma, urinary incontinence, erectile dysfunction, diabetes and neurodegenerative disorders (Ahmed et al., 2015; Dong et al., 2016). These effects have been linked to both its established properties, but also non-canonical effects including anti-oxidative and anti-inflammatory effects. In particular, NIC has been demonstrated to regulate the NF-κB and p38 MAPK in several disease models (He et al., 2019a; Khames et al., 2019a). Since NF-κB and MAPK signaling are vital in the formation of osteoclast, we hypothesized that NIC could potentially exert biological effects against RANKL-induced osteoclast differentiation and/or bone resorption.

Thus, in this study, complementary *in vitro* cellular and biochemical assays was carried out to investigate these effects. Additionally, using the *in vivo* murine model of ovariectomy (OVX)-induced bone loss, we further established the therapeutic benefits of NIC administration against osteoclast-mediated bone destruction. The OVX bone loss model is often adopted to mimic the conditions of post-menopausal osteoporosis and for investigating the biological effects of pharmacological agents. We found that NIC effectively inhibited RANKL-induced osteoclast formation and bone resorption, at least in part *via* the attenuation of early RANKL-induced activation of NF-κB and p38 signaling cascades. This consequently hampered the downstream induction of c-Fos and NFATc1, and osteoclast gene expression. The *in vivo* administration of NIC was also found to alleviate the effects of OVX-induced bone loss by suppressing osteoclast formation and bone resorbing activity.

## Materials and Methods

### Regents and Antibodies

Nicorandil (NIC) purchased from Sigma-Aldrich (St. Louis., MO, United States) was dissolved in 100% DMSO to a concentration of 50 mM and stored at −20°C for subsequent use. The NIC solution was retrieved from storage and further diluted to desirable working concentrations for culturing media. Asset recombinant murine macrophage-colony stimulating factor (M-CSF) and receptor activator of nuclear factor-κB (NF-κB) ligand (RANKL) were obtained from R&D Systems (Minneapolis, MN, United States). Minimal Essential Medium Eagle–Alpha Modification was procured from Shanghai Basalmedia Technologies Co., Ltd. (Shanghai, China). Fetal bovine serum (FBS) and penicillin/streptomycin were sourced from Gibco (Thermo Fisher Scientific, Waltham, MA, United States). The Cell Counting Kit-8 (CCK-8) was procured from Dojindo Molecular Technologies, Inc. (Kumamoto, Japan) and the TRAP staining kit from Sigma-Aldrich (St. Louis, MO, United States). The PrimeScript RT Reagent Kit and SYBR® Premix Ex Taq™ were bought from Takara Bio, Shiga, Japan. Specific primary antibodies against GAPDH, phospho-ERK (p-ERK), total ERK, phospho-p38 (p-p38), total p38, phospho-p65 (p-p65), total p65, IκBα and associated secondary antibodies were purchased from Cell Signaling Technology (Danvers, MA, United States). A specific primary antibody against the nuclear factor of activated T-cells 1 (NFATc1) was purchased from Absin Bioscience Inc. (Shanghai, China).

### Isolation of Primary Murine Bone Marrow Macrophages and Bone Marrow-Derived Stroma Cells

Six-week-old C57/BL6 male mice were sacrificed under anesthesia with 1% pentobarbital (40 mg/kg body weight). Long bones were rapidly separated, and then we isolated the primary murine BMMs and BMSCs through marrow flushing of the long bones. The isolation and culture of bone mesenchymal stem cells (BMSCs) were prepared as previously described (Xu et al., 2018). The extracted BMMs were cultured in α-MEM with 30 ng/ml M-CSF (complete α-MEM), 10% heat-inactivated FBS, and 100 U/ml penicillin-streptomycin (Xie et al., 2018). BMSCs were maintained in α-MEM with 100 U/ml penicillin-streptomycin and 15% FBS. Both cells were maintained at 37°C with a moist ambient in a 5% CO_2_ incubator.

### *In Vitro* Osteoclastogenesis Assay

Osteoclastogenesis assay was applied to examine the *in vitro* effects of NIC on osteoclast formation. Multinucleated osteoclasts were generated from BMM precursor cells as follows; 1 × 10^4^ BMMs/well were incubated in triplicates in 96-well plates and reincubated in a complete α-MEM for at least 18 h; The next day, cells were stimulated with 50 ng/ml RANKL without (positive control) or with various concentrations of NIC (25, 50, 100, or 200 μM). Culture media containing M-CSF, RANKL, and NIC were replenished every other day until large well-spread multinucleated osteoclasts were observed on day 5 in groups treated with RANKL only. At this stage, the cells were gently washed with PBS, fixed in 4% paraformaldehyde (PFA) for 20 min, and then stained for tartrate-resistant acid phosphatase (TRAP) activity (Chen et al., 2019). Phase-contrast images were captured on a digital camera-equipped Olympus light microscope (Olympus Life Science, Tokyo, Japan). The number and cell-spread area of TRAP-positive multinucleated osteoclasts with either three or more nuclei were quantified using ImageJ software [National Institute of Health (NIH), Bethesda, MD, United States]. The experiment was repeated in triplicate.

### Cell Viability Assay

Cellular toxicity of NIC on BMMs were assessed using the CCK-8 cell proliferation/cytotoxicity assay in accordance with manufacturer’s protocol. Briefly, BMMs seeded at a density of 8 × 10^3^ cells/well in 96-well plates in triplicates in complete α-MEM for 24 h were incubated with increasing concentrations of NIC (6.25, 12.5, 25, 50, 100, 200, or 400 μM) for 48, 72, and 96 h. The final stages of the experiment involved the re-incubation of cells with 10 µl of CCK-8 reagent for further 2 h after which we determined the absorbance or optical density (OD) on a microplate absorbance spectrophotometer at 450 nm. The experiment was repeated in triplicate.

### *In Vitro* Bone Resorption Assay

Herein, we evaluated the effect of NIC on osteoclast base on bone resorption assay. BMMs were incubated onto the bone-mimicking hydroxyapatite-coated OsteoAssay Stripwell Plates (Corning, NY) at a density of 1 × 10^4^ cells/well in triplicates for each experimental condition in a complete α-MEM. Cells were stimulated in 50 ng/ml RANKL without (positive control) or with different concentrations of NIC (12.5, 25, 50, or 100 μM) for 5 days. Culture media containing M-CSF, RANKL, and NIC were replenished daily. On fifth day, cells were treated with 6% sodium hypochlorite solution and then gently washed with PBS for twice. Phase-contrast images were captured on a digital camera-equipped Olympus light microscope (Olympus Life Science, Tokyo, Japan). The number and cell-spread area of TRAP-positive multinucleated osteoclasts with three or more nuclei were quantified using ImageJ software [National Institute of Health (NIH), Bethesda, MD, United States]. The experiment was repeated at least three times.

### RNA Extraction and Real-Time Quantitative PCR Analyses

The effects of NIC on osteoclast marker gene expression was assessed through real-time quantitative PCR analyses. BMMs incubated at a density of 8 × 10^3^ cells/well in 96-well plates (in triplicates) in the detailed α-MEM were stimulated with 50 ng/ml RANKL without (positive control) or with different concentrations of NIC (0, 12.5, 25, or 50 μM) for 5 days. The Oxygen RNA Miniprep Kit (Oxygen, Union City, CA, United States) was used to extract total RNA from each group based on the manufacturer’s instructions. The PrimeScript RT Reagent Kit and 1 μg of extracted RNA templates were used for reverse transcription and in the generation of cDNA. The resulting cDNA was used as templates in real-time qPCR reaction mixtures containing SYBR^®^ Premix Ex Taq^TM^ and specific primers as shown in Table 1 qPCR reactions were performed on an ABI 7500 Real-Time PCR System (Applied Biosystems, Foster City, CA, United States) with the following cycling parameters: 30 cycles of 96°C for 5 s, and 58°C for 30 s, and 72°C for 20 s (amplification); and a final extension at 72°C for 90 s. We adopted the comparative 2^−ΔΔCT^ method to normalize the relative expression levels of each gene against the *GAPDH* housekeeping gene. All reactions were performed in triplicates.

**TABLE 1:** Primer pairs sequences against osteoclast genes used in qPCR.

Gene	Forward 5ʹ → 3ʹ	Reverse 5ʹ → 3ʹ
*Nfatc1*	TGC​TCC​TCC​TCC​TGC​TGC​TC	GCA​GAA​GGT​GGA​GGT​GCA​GC
*cFos*	CCA​GTC​AAG​AGC​ATC​AGC​AA	AAG​TAG​TGC​AGC​CCG​GAG​TA
*Ctsk*	CTT​CCA​ATA​CGT​GCA​GCA​GA	TCT​TCA​GGG​CTT​TCT​CGT​TC
*Trap*	CAA​AGA​GAT​CGC​CAG​AAC​CG	GAG​ACG​TTG​CCA​AGG​TGA​TC
*Dcstamp*	AAA​ACC​CTT​GGG​CTG​TTC​TT	AAT​CAT​GGA​CGA​CTC​CTT​GG
*Gapdh*	GGT​GAA​GGT​CGG​TGT​GAA​CG	CTC​GCT​CCT​GGA​AGA​TGG​TG

### Protein Extract and Immunoblotting

Western blot analyses were used to investigate the effect of NIC from the beginning to end of RANKL signaling events. During the initial RANKL signaling events, 5 × 10^5^ of BMMs/well were seeded in 6-well plates with complete α-MEM for 24 h and pre-treated without or with 25 μM NIC (in serum-free α-MEM containing 30 ng/ml M-CSF) for 2 h followed by its stimulation with 50 ng/ml RANKL for 10, 20, 30, and 60 min. In the assessment of terminal RANKL signaling events, 3 × 10^5^ of BMMs/well were seeded in 6-well plates with complete α-MEM and stimulated with 50 ng/ml RANKL without or with 25 μM NIC for 1, 3, and 5 days. Unstimulated BMMs acted as negative controls. In the final stage of the experimental period, radioimmunoprecipitation assay (RIPA) lysis buffer (Beyotime Biotechnology, Jiangsu, China) containing phosphatase/protease inhibitor cocktail (Sigma-Aldrich) was used to extract the total cellular proteins. The cell lysates were subsequently cleared *via* centrifugation at 12,000 × g for 15 min. The resultant protein supernatants were retained. The protein concentrations were quantified using the bicinchoninic acid (BCA) assay following the manufacturer’s protocol. Proteins were separated by 10% sodium dodecyl sulfate-polyacrylamide gel electrophoresis (SDS-PAGE) and transferred to polyvinylidene difluoride membranes (Millipore, Bedford, MA). Membranes were then blocked in 5% (w/v) skim milk in Tris-buffered saline-Tween 20 (TBST) for 1 h at room temperature followed by their incubation with specific primary antibodies (diluted in 1% (w/v) skim milk-TBST) against GAPDH (1:1,000), p-p38 (1:1,000), p38 (1:1,000), p-ERK (1:1,000), ERK (1:1,000), NFATc1 (1:1,000), p-p65 (1:1,000), p65 (1:1,000), IκBα (1:1,000), and c-Fos (1:1,000) overnight at 4°C with gentle agitation. Membranes were extensively washed with TBST and re-incubated with appropriate HRP-conjugated secondary antibodies [diluted in 1% (w/v) skim milk-TBST] for 1 h at room temperature. The protein-antibody was subjected to a chemiluminescence substrate to detect its reactivity on an Odyssey Fc Imaging System (LI-COR Biosciences, Lincoln, NE, United States).

### Nuclear Translocation of p-p65

To explore the change of NF-κB nuclear translocation after NIC treatment, 1 × 10^5^ BMMs/well were seeded in 6-well plates on 2-mm glass coverslips in complete α-MEM (α-MEM containing 10% FBS and 30 ng/ml M-CSF) and treated with or without 25 μM NIC for 24 h. The coverslips were collected, washed twice with PBS, fixed in 4% paraformaldehyde for 10 min, and resolved with 0.1% Triton X-100 (Sigma, United States) for 10 min. Nonspecific binding of was blocked by Goat serum (4%) for 1 h. After washed glass coverslips twice with PBS, cells were incubated with p-p65 primary antibodies (CST, Danvers, MA, United States; dilution 1:100) in TBST at 4°C. The fluorescent secondary antibodies were used to visualize the relevant subsets after 12 h. After 1 h, cells were washed twice with PBS and then stained in 4,6-diamidino- 2-phenylindole (DAPI) for 5 min and examined under a confocal fluorescence microscope (Leica TCS-SP5, DM6000-CFS).

### Osteoblast Differentiation and Mineralization Function Assays *In Vitro*

To examine the effects of NIC on osteoblast differentiation and function, primary mouse BMSCs were seeded into 48-well plates at a density of 8 × 10^4^ cells/well where they grew to 90% confluence. BMSCs were stimulated with osteogenic media containing 10 mM β-glycerophosphate, 50 μg/ml ascorbic acid, and 10^−7^ mM dexamethasone without or with various concentrations of NIC (12.5, 25, or 50 μM). Half of the osteogenic medium was replenished every 2 days. BMSCs were stimulated under osteogenic conditions for 7 days and the cells fixed in 4% PFA before stained for alkaline phosphatase (ALP) (Beyotime Biotechnology) activity to determine the effects of NIC on osteoblast differentiation. On the other hand, BMSCs were cultured under osteogenic conditions for 21 days and the cells fixed in 4% PFA were washed three times with 70% ethanol and stained with 1% Alizarin Red S (ARS) solution for 30 min (Beyotime Biotechnology) to determine the effects of NIC on osteoblast mineralization function. ImageJ software (NIH) was adopted to evaluate the ALP and mineralization activity of osteoblast.

### *In Vivo* Murine Model of Ovariectomy-Induced Bone Loss

All animal experiments and surgical procedures were approved by the Animal Care and Experiment Committee of Shanghai Jiao Tong University School of Medicine. The study was conducted following the Guide for the Care and Use of Laboratory Animals of the National Institute Health (United States). Twenty-four female C57/BL6 mice (7-week-old; 20–25 g) were obtained and housed in the Department of Laboratory Animal Science at Shanghai Ninth People’s Hospital. All mice were kept in a temperature control environment of 22–25°C and 60 ± 5% humidity with 12 h of light and dark cycles. All mice were fed on standard rodent feed and a sufficient supply of fresh water. After 1 week of acclimatization, the mice were randomly categorized into four groups (*n* = 6 mice per group): (Rachner et al., 2011) Sham control + vehicle (sham-operated with PBS injections); (Harvey et al., 2010) OVX + vehicle (with PBS injections); (Yedavally-Yellayi et al., 2019a) OVX + low dose NIC (3 mg/kg body weight/day); and (Del Puente et al., 2015) OVX + high dose NIC (6 mg/kg body weight/day). The dose of nicorandil used was as previously reported (Ahmed et al., 2011). Mice were anaesthetized with intraperitoneal injections of 1% pentobarbital (40 mg/kg body weight) and received either sham or bilateral ovariectomy to remove the ovaries *via* back incisions. An equal weight of adipose tissue was excised from the sham-operative mice. NIC administration commenced 1-week after surgery to enable post-operative recovery. NIC was administered by oral gavage once daily for 8 weeks. At the end of the 8-weeks experimental period, all mice were euthanized by overdosing with 1% pentobarbital injection. Then, whole tibias and femurs were excised, cleaned of soft tissues, and then fixed in 4% PFA in preparation for downstream micro-computed tomography (cT) and histological assessments.

### Micro-cT Scanning

The fixed distal femur specimens were subjected to a micro-CT analysis on a μCT 40 desktop cone-beam system (SCANCO Medical AG, Brüttisellen, Switzerland). Images were acquired using an X-ray energy set at 70 kV, 114 μA, and scanning isotropic resolution of 10 μm with a fixed exposure time of 300 ms. Quantitative morphometric measurements were implemented within a defined square region of interest 0.5 mm below the femoral growth plate. Quantitative morphometric parameters analyzed included the percentage bone volume fraction (BV/TV; %), trabecular number (Tb.N; mm^−1^), trabecular thickness (Tb.Th; mm), and trabecular separation (Tb.Sp; mm).

### Histological and Histomorphometric Assessment

The fixed tibial bones were decalcified in 10% EDTA for 2 weeks and embedded into paraffin blocks before cut into 5 μm thick slices. The slices were stained with hematoxylin and eosin (H&E) for TRAP activity. Stained histological sections were imaged under an Olympus light microscope equipped with a high-resolution digital CCD camera. ImageJ software (NIH) was used to quantify the number of TRAP-positive multinucleated cell per bone surface (N.Oc/BS).

### Statistical Analyses

All data were compared among multiple groups by one-way analysis of variance (ANOVA), SPSS 22.0 software (IBM Corporation, New York, NY, United States) was applied to perform the Students *t*-test and one-way analysis of variance (ANOVA) for multifactorial comparisons in this study. Presented as the mean ± SD or representative images from three or more independent experiments. *p*-values ≤ 0.05 (*), ≤ 0.01 (**), and ≤ 0.01 (***)were considered statistically significant, highly statistically signifificant, and extremely statistically signifificant.

## Results

### NIC Inhibits *In Vitro* Osteoclast Formation in Response to RANKL Stimulation

**FIGURE 1:**
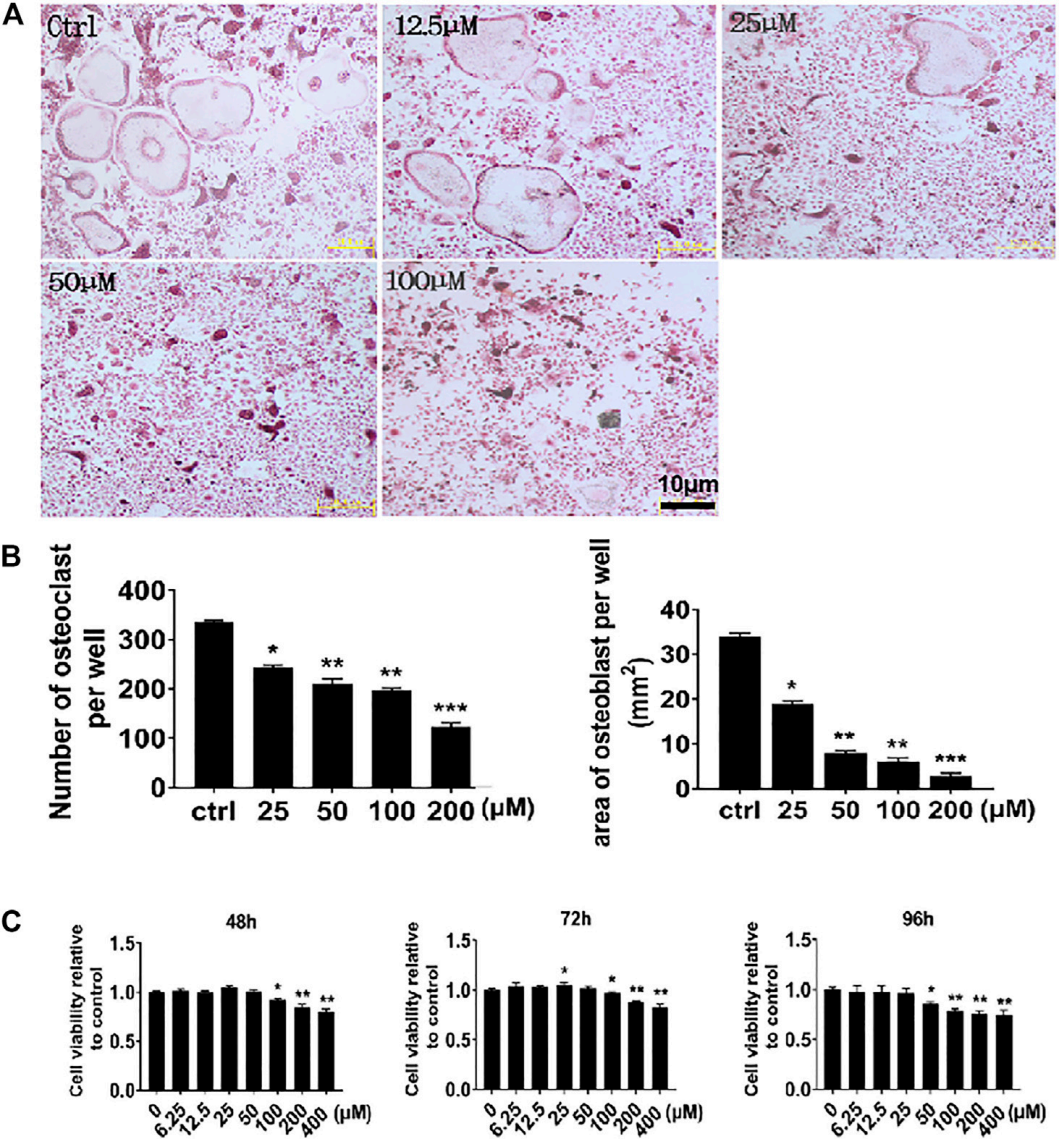
NIC suppresses the formation of osteoblasts *in vitro* in response to RANKL stimulation. **(A)** The BMMs that M-CSF relies on are stimulated with 50 ng/ml RANKL (positive control) or NIC (12.5, 25, 50 or 100 μM) at different concentrations for 5 days, and then the cells were treated with 4% paraformaldehyde fix it and stain it for TRAP. **(B)** Quantitative analysis of the total number of TRAP-positive multinucleated osteoblasts with three or more nuclei. **(C)** The viability of BMM cells was assessed by the CCK-8 assay after treated with different concentration of the NIC in the presence of 30 ng/ml M-CSF, concentrations of NIC up to 50 μM for 48 and 72 h, and up to 25 μM for 96 h did not show cytotoxic effects. All experiments were carried out independently at least three times, independently (*p < 0.05, **p < 0.01, ***p < 0.001, ****p < 0.0001). BMMs, Bone marrow-derived macrophages; RANKL, receptor activator of nuclear factor kappa B ligand; TRAP, tartrate-resistant acid phosphatase.

We first examined the *in vitro* effects of NIC against the ability of mononuclear precursors to form multinucleated osteoclasts in response to RANKL stimulation. Thereafter, M-CSF-dependent BMMs were stimulated with RANKL without or with suggested concentrations of NIC for 5 days followed by the assessment of multinucleated osteoclast formation through staining for TRAP activity. As shown in Figures 1A,B, stimulation with RANKL only (Ctrl) without co-treatment with NIC resulted in the formation of numerous large and well-spread TRAP + ve multinucleated osteoclasts. In contrast, co-treatment with NIC dose-dependently inhibited RANKL-induced osteoclast formation with many cells remaining as mononuclear TRAP + ve precursor cells. To investigate whether the inhibitory effect of NIC was due to cytotoxic effects against BMMs we carried out CCK-8 cell viability assay. BMMs were treated with indicated concentrations encompassing those used in the osteoclast formation assay for 48, 72, and 96 h. As demonstrated in Figure 1C, concentrations of NIC up to 50 μM for 48 and 72 h, and up to 25 μM for 96 h did not show cytotoxic effects. On the other hand, high concentrations of NIC (>100 μM) significantly inhibited BMMs viability. Therefore, these results suggested that NIC inhibit the *in vitro* formation of osteoclast in a dose-dependent manner, whereas higher concentrations (>100 μM) had moderate levels of cytotoxicity.

### NIC Suppressed RANKL-Induced Gene Expression

**FIGURE 2:**
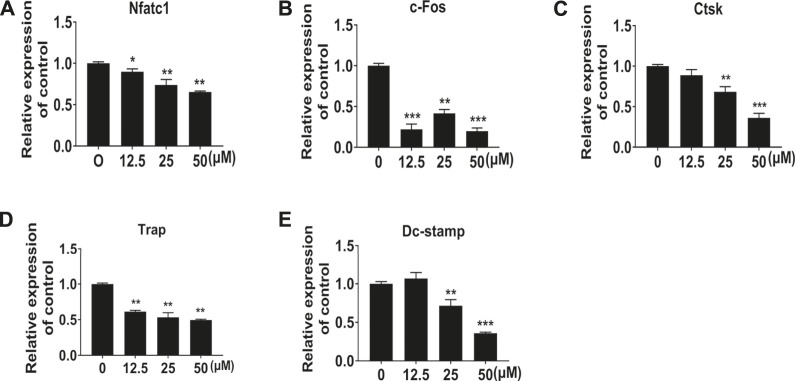
NIC suppressed RANKL-induced gene expression In the presence of 0, 12.5, 25, and 50 μM NIC, BMMs were treated with M-CSF (30 ng/ml) and RANKL (50 ng/ml) for 5 days. Real-time quantitative PCR to analyze the expression of several types of osteoblast marker genes, including Nfatc1, c-Fos, Ctsk, Trap, and Dc-stamp. Data were normalized against Gapdh mRNA and expressed as fold induction relative to treatment with vehicle control, which was assigned an average value of 1. Data are presented as the mean ± SD compared to control (*p < 0.05, **p < 0.01, ***p < 0.001). All experiments were carried out independently at least three times.

To further demonstrate the inhibitory effect of NIC on osteoclast differentiation and formation, real-time quantitative PCR was used to examine the expression of several types of osteoblast marker genes. In line with the cellular effects, the expression of osteoclast marker genes significantly reduced after NIC treatment (Figures 2A–E). Genes encoding proteins refer to precursor differentiation and formation such as transcription factors c-Fos and NFATc1 and the precursor cell fusion factor DC-STAMP were markedly suppressed with NIC. Comparably, the expression of genes encoding enzymes involved in bone resorption such as TRAP and CTSK was significantly lessened. Thus, the decline in osteoclast gene expression provides further evidence that NIC inhibits osteoclast formation *in vitro*.

### NIC Reduces Osteoclastic Bone Resorption *In Vitro*

**FIGURE 3:**
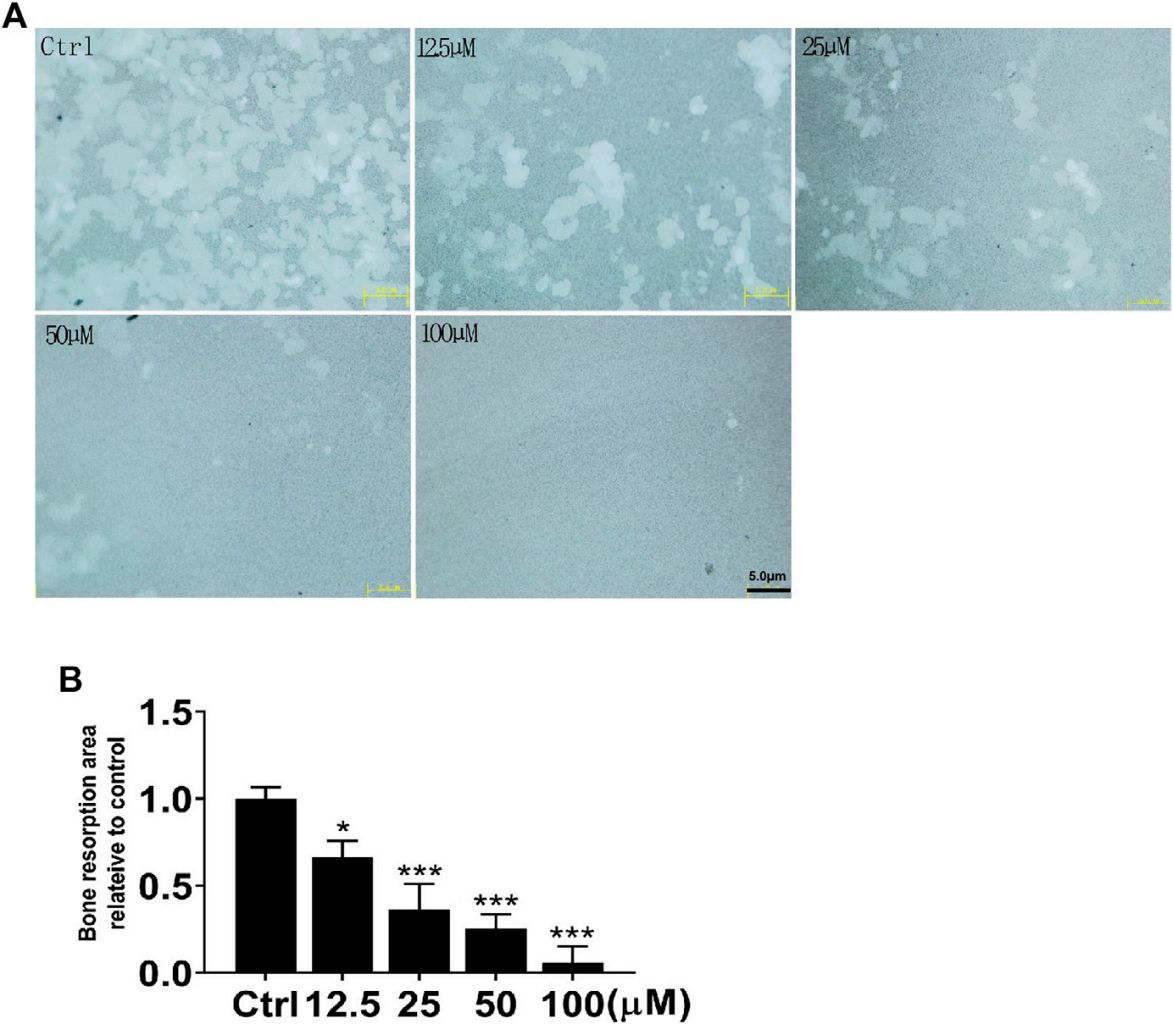
NIC attenuates osteoclastic bone resorption *in vitro*. BMM-derived osteoclasts were cultured with bone-mimicking calcium phosphate-coated Osteo Assay Stripwell plates. After removing the osteoclasts with sodium hypochlorite, Bone resorption dents were captured using the citation three Cell Imaging Reader. The total area of bone resorption under each treatment standard is quantitatively analyzed, and it is shown as the ratio of comparison with only RANKL treatment. All experiments were carried out independently at least three times (*p < 0.05, **p < 0.01, ***p < 0.001). BMMs, Bone marrow-derived macrophages; RANKL, receptor activator of nuclear factor kappa B ligand.

Following the identification of the NIC inhibitory effect on osteoblasts, we further investigated the NIC effect on osteoclast function, particularly on bone resorption. Of note, osteoclasts were generated *via* stimulation of BMMs cultured on bone-mimicking hydroxyapatite-coated OsteoAssay Stripwell plates with RANKL in the absence or presence of indicated concentrations of NIC. Cultured cells were removed after 5 days to examine the bone resorption dents. The number and area of bone resorption dents of cells treated with NIC lessened in a dose-dependent manner (Figures 3A,B). Treatment with high concentrations of NIC particularly 100 μM completely abolished osteoclastic bone resorption. However, treatment with 12.5 and 25 μM of NIC reduced bone resorption by approximately 35 and 65%, respectively relative to RANKL only controls (Figure 3B). Therefore, our results suggested that thus NIC not only inhibits osteoclast formation, but also attenuates osteoclastic bone resorption.

### NIC Inhibit the RANKL-Induced Activation of NF-κB and p38 Signaling Pathways

**FIGURE 4:**
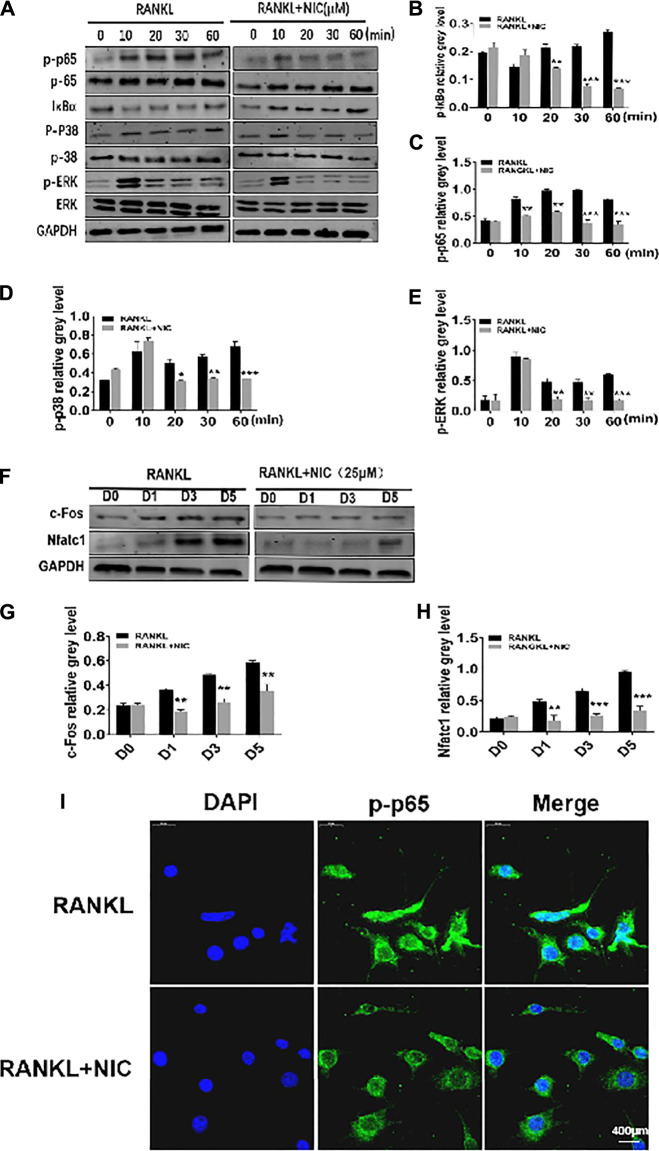
NIC curbed the RANKL-induced activation of NF-κB and p38 signaling pathways. **(A)** NIC blocked RANLK-induced IκBαdegradation and consequently obstructed phosphorylation of p65, p38 and ERK phosphorylation. **(B–E)** Quantitative analysis of IκBα, p65 phosphorylation, p38 Phosphorylation and ERK phosphorylation expression. **(F)** NIC treatment inhibited the expression of c-Fos and NFATc1 signaling pathways. **(G–H)** Quantitative analysis of the expression of c-Fos and NFATc1. **(I)** To detect NF-κB nuclear translocation, p-p65 was examined by immunostaining. All experiments were performed independently at least three times. The data are presented as the mean ± SD (*p < 0.05, **p < 0.01, ***p < 0.001).

To better drive multinucleated osteoclast formation from mononuclear pre-cells in response to RANKL, various signaling pathways are essential for coordinated and timely activation. Herein, through Western blot analyses, we examined the initial stage of two pivotal RANKL-responsive signaling cascades, NF-κB and MAPK to determine the potential mechanism by which NIC exerts its inhibitory effects against osteoclast formation. In the NF-κB signaling cascade, RANKL stimulation induces the rapid dissolution of IκBα from 5 to 30 min and back to basal levels by 60 min (Figures 4A,B). This was concomitantly associated with elevated p65 phosphory-lation status (Figures 4A,C), an event that is necessary for the nuclear translocation of p65. These two inter-related events are required in the initiation and activation of NF-κB transcriptional activity. Conversely, treatment with 25 μM NIC blocked RANLK-induced IκBα degradation and consequently obstructed p65 phosphorylation (Figures 4A–C). Similarly, RANKL stimulation rapidly induced the phosphorylation of MAPK members p38 and ERK. However, NIC treatment repressed p38 phosphorylation and ERK phosphorylation (Figures 4A,D,E). Therefore, our data suggest that the inhibitory effect of NIC is partly dependent on the inhibition of RANKL-induced p38 MAPK and NF-κB signaling activation.

Early activation of MAPK and NF-κB is essential for efficient induction of the two important downstream transcription factors, c-Fos and NFATc1. c-Fos induction drives mononuclear precursor cell down the osteoclast lineage with other transcription factors (such as NF-κB) to regulate the robust induction of NFATc1. NFATc1 transcription regulates the expression of numerous osteoclast genes to initiate most distal transcription necessary for precursor cell fusion and end-stage of osteoclast differentiation including those analyzed in Figure 2. Figures 4F–H shows that stimulation of RANKL induces a significantly robust and time-dependent induction of both c-Fos and NFATc1 over the course of the 5 days of osteoclast formation. In stark contrast, treatment of cells with NIC markedly abrogated the upregulation of both transcription factors. In addition, through immunofluorescence, RANKL was revealed to induce the translocation of p-p65 into the nucleus, whereas NIC inhibited the translocation of p-p65 into the nucleus (Figure 4I). These results suggest that anti-osteoclastic effects of NIC partly depend on the suppression of initial RANKL-induced activation of NF-κB and p38 MAPK signaling which consequently hampered the downstream induction of NFATc1 and c-Fos.

### NIC did not Affect Osteoblast Differentiation and Mineralization Function

**FIGURE 5:**
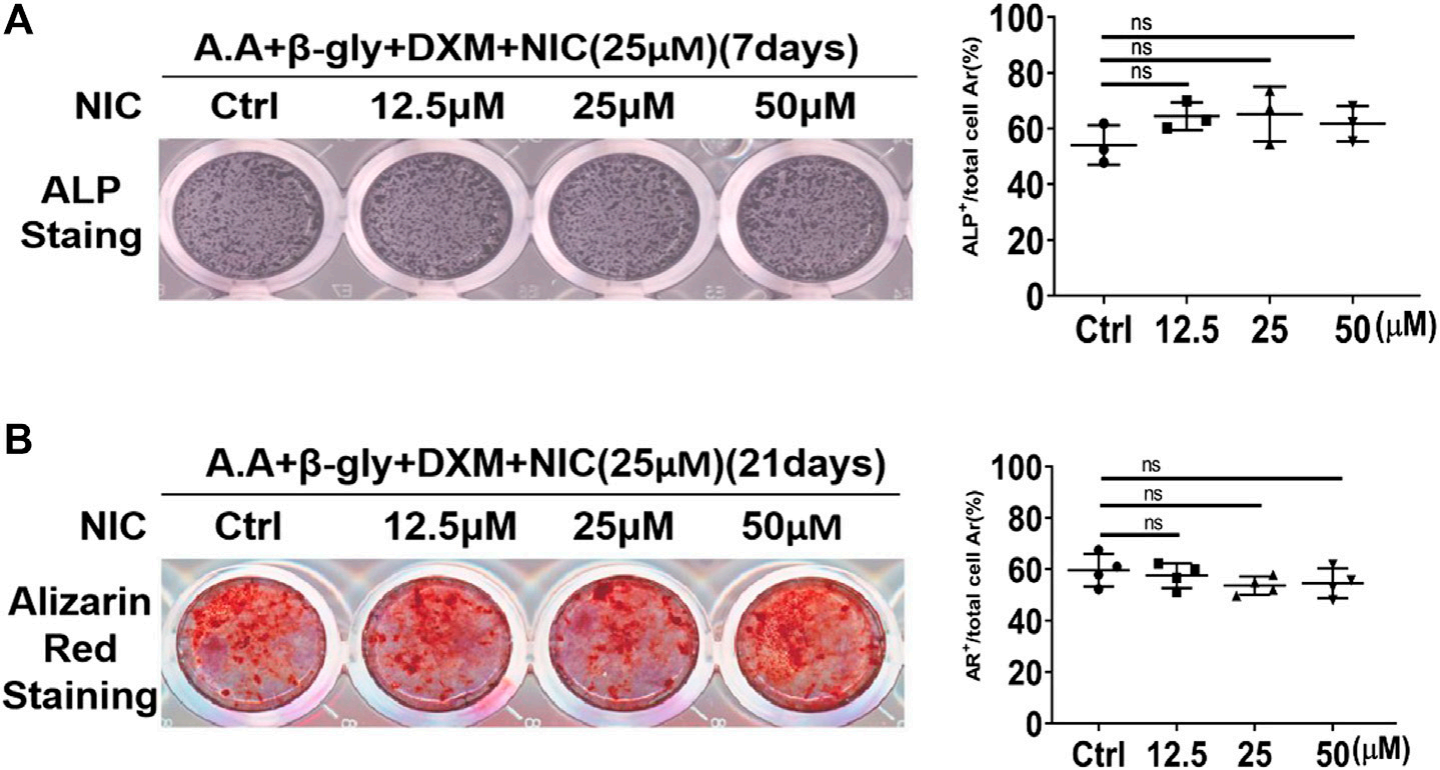
NIC does not affect osteoblast differentiation and mineralization function *in vitro*. Stimulated with ascorbic acid (A.A), dexamethasone (DXM) andβ-glycerophosphate (β-Gly), BMSCs were treated with NIC (0, 12.5, 25, and 50 μM) for 7, 21 days. The ALP (Alkaline phosphatase) and Alizarin Red Staining indicated no significant difference between control and treated groups. The data are presented as the mean ± SD (*p < 0.05, **p < 0.01). All experiments were carried out independently at least three times.

Osteoclasts and osteoblasts activities play an important role in the mediation of bone homeostasis. Preliminary studies showed that NIC inhibits the formation and function of osteoclast. Therefore, we further explored whether NIC could also exert biological effects against bone-forming osteoblasts. Extracted primary BMSCs were cultured under osteogenic conditions without or with the same concentrations of NIC used during osteoclast assays. After 7 or 21 days of osteogenic differentiation, cells were fixed and stained for alkaline phosphatase (ALP) activity and mineralized nodule formation (Alizarin Red S staining; ARS), respectively. We found that NIC did not affect the differentiation of primary osteoblast (ALP; Figure 5A) and mineralized bone nodule formation (ARS; Figure 5B). Thus, the result showed that the concentration of NIC did not enhance or inhibit osteoblast differentiation and bone formation activity.

### *In Vivo* Administration of NIC Protects Mice Against Ovariectomy-Induced Bone loss

**FIGURE 6:**
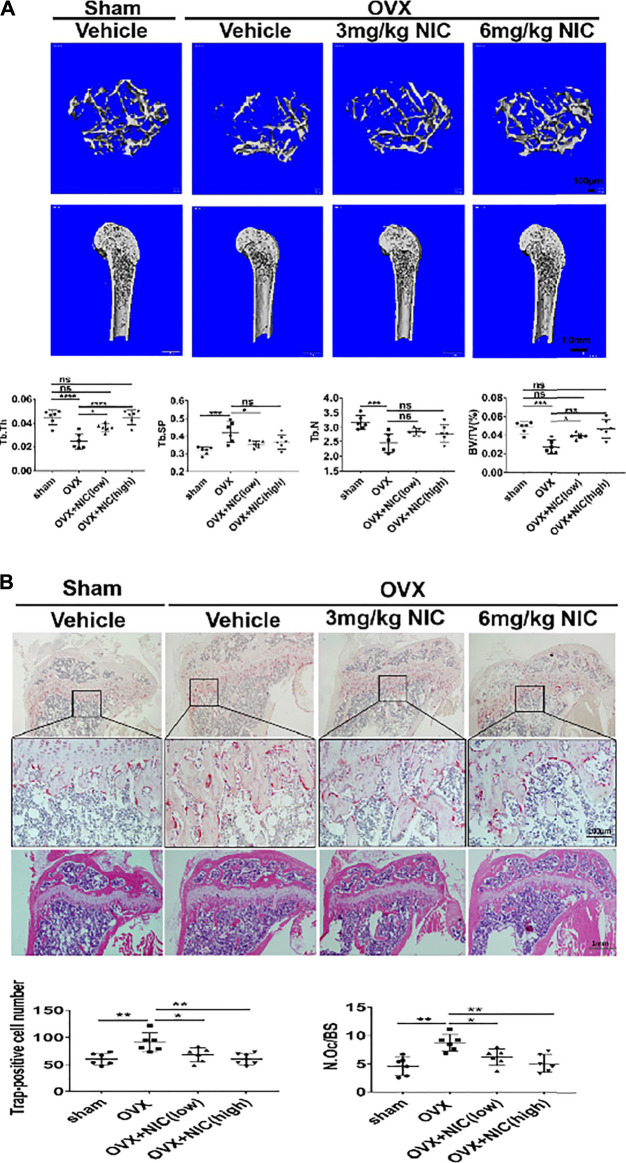
*In vivo* administration of NIC protects mice against ovariectomy-induced bone loss. **(A)** μ-CT scanning and 3D reconstructed images of distal femurs (coronal and axial planes) and cortical bone from the femoral midshaft showed that bone loss was attenuated by administering NIC. Quantitative analyses of bone morphometric parameters of OVX mice was consistent with an osteoporotic phenotype showing significant reductions in bone volume (BV/TV), trabecular number (Tb. N), and trabecular thickness (Tb. Th), with increased trabecular spacing (Tb. Sp). Data are presented as mean ± SD. (*p < 0.05, **p < 0.01, ***p < 0.01, compared with the OVX group). **(B)** Histological examination of the proximal tibial bone sections provided additional evidence for the osteoprotective effect of NIC against OVX-induced bone loss. Compare with the NIC treated group, the OVX exhibited a plentiful TRAP-positive osteoclast present in the osteolytic lesion. All experiments were carried out independently at least three times (*p < 0.05, **p < 0.01, ***p < 0.01).

The *in vitro* studies have clarified the inhibitory effect of NIC on RANKL-induced osteoclasts formation and function. The OVX model is commonly used to mimic post-menopausal osteoporosis. During this stage, mice that underwent OVX were daily administered with either vehicle (PBS), or NIC at 3 mg/kg (low-dose) or 6 mg/kg (high-dose) for 8-weeks. Based on the 3D reconstructions of the distal femur, OVX mice that received vehicle (PBS) for the 8-weeks treatment period had significant trabecular bone loss than the sham-operated control mice (Figure 6A). Conversely, treatment with NIC dose-dependently protected mice (low-dose and high-dose) from the deleterious bone effects of OVX. Quantitative analyses of bone morphometric parameters of OVX mice were consistent with their osteoporotic phenotype whereby significant reductions in bone volume (BV/TV), trabecular number (Tb.N), and trabecular thickness (Tb.Th), with enhanced trabecular spacing (Tb.Sp) were observed. On the other hand, NIC treatment protected mice from OVX-induced bone loss, improved BV/TV, Tb.N, Tb.Th, and Tb.Sp in a dose-dependent manner (Figure 6A).

Histological examination of the proximal tibial bone slices provided additional evidence for the osteoprotective effect of NIC against OVX-induced bone loss. For instance, the H&E stained slices had less bone loss in NIC treated mice whereas TRAP stained sections showed marked reductions in TRAP + ve cells lining the trabecular bone surface (Figure 6B). According to the histomorphometric analyses, treatment of OVX mice with high-dose (6 mg/kg body weight/day) of NIC significantly reduced the total number (N.Oc) and activity (osteoclast surface/bone surface; Oc. S/BS) of TRAP + ve osteoclasts on the trabecular bone surface in OVX mice following NIC treatment. Collectively, our *in vitro* cellular and biochemical analyses coupled with *in vivo* bone loss model demonstrated the potential of NIC in the management and/or treatment of osteoclast-mediated osteolytic conditions including postmenopausal osteoporosis.

## Discussion

Bone homeostasis is maintained by the dynamic balance between bone resorption and bone formation mediated by osteoblasts and osteoclast, respectively. Perturbations in the bone homeostasis balance can lead to excessive formation of osteoclast and bone resorption which then transpire to many osteolytic bone diseases such as osteoporosis (Kular et al., 2012; Kim et al., 2014; Langdahl et al., 2016; Katsimbri, 2017; Myneni and Mezey, 2017; Zhu et al., 2017; Yedavally-Yellayi et al., 2019b). Current therapeutic interventions strategies, including selective estrogen receptor modulators, estrogen replacements and anti-RANKL antibody (Denosumab), and bisphosphonates have demonstrated beneficial effects on osteoclast-mediated osteolysis. However, their long-term clinical application is associated with serious adverse reactions for instance cardiovascular and cerebrovascular events, osteonecrosis of the jaw, malignant tumor formation, nephrotoxicity, and atypical fractures (McGreevy and Williams, 2011; Cheng et al., 2019). As a result, a new therapeutic strategy with high efficiency and few side effects are needed for the treatment of osteoclast-mediated bone disorders are urgently needed.

In this research, we showed that Nicorandil (NIC) exerts anti-osteoclastic and anti-resorptive effects *in vitro* and *in vivo*. Our *in vitro* based cellular assays showed that NIC inhibited RANKL-induced osteoclast formation and attenuated osteoclast bone resorption. Biochemical analyses further suggest that the inhibitory effect of NIC is partly attributable to the initial activation of NF-κB and p38 MAPK signaling pathways which consequently mitigate the downstream induction of transcription factors c-Fos and NFATc1. Lack of NFATc1 induction resulted in downregulated expression of key osteoclast genes encoding proteins involved in the osteoclast division and bone resorption (Asagiri et al., 2005). Our vivo study showed that the administration of NIC could protect mice from OVX-induced bone loss by suppressing osteoclast-mediated bone resorption. Overall, our results revealed NIC as a promising therapeutic agent for the treatment/management of osteoclast-mediated osteolytic diseases including osteoporosis. NIC as a vasodilatory drug against angina also exhibited a cardioprotective effect which is attributed to its K_ATP_ channel-activating and nitrate/nitric oxide (NO)-like properties (Sahara et al., 2012; He et al., 2018). Previous reports have shown that NO production *via* NO synthase (NOS) in response to RANKL in osteoclasts inhibits osteoclast division and bone resorption *in vivo* and *in vitro.* Hence, this is an important negative feedback loop that limits excessive osteoclast formation and activity (Zheng et al., 2006). A brief report by Iwaki and colleagues on cellular-based and inhibition recovery assays showed that NIC *via* its K_ATP_ channel-activating and nitrate/nitric oxide (NO)-like properties suppressed the *in vitro* differentiation of osteoclasts (Iwaki et al., 2016). Similarly, we reported similar findings in our *in vitro* osteoclast formation assay within the same concentration range as that used by Iwaki and colleagues and found extended the inhibitory effect of NIC to include osteoclastic bone resorption. Further, we demonstrated that the concentration of NIC that exerted inhibitory effects against osteoclast formation and function did not adversely affect osteoblast formation or mineralized bone nodule formation. Also, our study explored other potential mechanistic insights of NIC inhibitory effect on osteoclast division and bone resorption. This is in light of reports suggesting that NIC potentially inhibit inflammatory signaling pathways including MAPK and NF-κB under various pathological conditions (Ye et al., 2017; Qiang et al., 2018; He et al., 2019b; Khames et al., 2019b). However, both NF-κB and MAPK signaling are crucial signaling pathways that are stimulated in response to RANKL stimuli in the initial stage of osteoblast division (Kobayashi et al., 2001; Blair et al., 2005; Park et al., 2017). The binding of RANKL to receptor RANK on mononuclear precursor cells recruit TRAFs (particularly TRAF6) that activates a cascade of downstream signaling events to facilitate proliferation, differentiation and fusion of the precursor. Of these, activation of NF-κB transcriptional activity is driven by the phosphorylation and proteasomal degradation of IκBα following RANKL stimulation. IκBs are NF-κB inhibitory protein that retains NF-κB subunits (p65 and p50 heterodimers) in the cytoplasm in an inactive state (Hayden and Ghosh, 2008; Vallabhapurapu and Karin, 2009). The loss of IκBα results in phosphorylation and nuclear translocation of NF-κB p65/p50 heterodimers and their combination with other co-transcriptional activators collectively regulate gene transcription (Franzoso et al., 1997; Hayden and Ghosh, 2004; Vaira et al., 2008; Xing et al., 2013).

Concurrent with the activation of NF-κB is the activation of the MAPK signaling cascade. The MAPK signaling triad of JNK, ERK and p38 are simultaneously activated by phosphorylation which in turn promotes their nuclear translocation and transcriptional activity (Matsumoto et al., 2004). All three MAPK members is required for the efficient osteoclast formation (Ikeda et al., 2004; Lee et al., 2016; Cong et al., 2017). Importantly, the initial activation of NF-κB and MAPK signaling is essential for the downstream induction of c-Fos and NFATc1. NFATc1 is a relatively remote transcription factor for osteoclast formation. Its overexpression alone sufficiently drives osteoclast formation without RANKL (Matsuo et al., 2004; Asagiri et al., 2005). This is because, NFATc1 transcription adjust the expression of various osteoclast genes including *DC-STAMP* involved in precursor cell fusion (Yagi et al., 2005), *TRAP* and *CTSK* and are key enzymes for bone resorption and *NFATc1* itself *via* an auto-amplification loop (Franzoso et al., 1997; Yagi et al., 2005; Fretz et al., 2008; Kim et al., 2008). Consistent with the cellular effects, NIC was found to attenuate the activation of NF-κB and p38 MAPK signaling cascades. Lack of induction of NFATc1 was associated with significantly abrogated expression of osteoclast genetic genes.

In conclusion, the present biochemical analysis provides further insight into the mechanistic actions of NIC, at least in part, due to the attenuation of early NF-κB and p38 MAPK activation, and the annulling of the downstream induction of NFATc1 and its transcriptional activity. However, the exact mechanisms of NIC effect on NFATC1 is unclear, We need further research to understand better other potential pathway.

Together with the previously described K_ATP_ channel-activating and nitrate/nitric oxide (NO)-like properties effectually inhibited the formation of osteoclast and bone resorption function (Iwaki et al., 2016; Kalyanaraman et al., 2017). By replicating these *in vitro* results to *in vivo* experiments*,* the administration of NIC markedly protected mice from the deleterious bone-loss effects of ovariectomy. Significant improvements in bone volume and trabecular bone architecture were observed, however, histological assessment further revealed a reduction in the numbers of osteoclast and activity on the bone surface following NIC treatment. Collectively, our *in vitro* and *in vivo* results demonstrated the potential of NIC in the therapeutic management and/or treatment of osteoclast-mediated skeletal conditions such as osteoporosis, but the transport and absorption of NIC and the mechanisms affects osteoclast remain to be studied *in vivo*.

## Data Availability

The original contributions presented in the study are included in the article/Supplementary Material, further inquiries can be directed to the corresponding authors.
